# Transposable Elements and Their Epigenetic Regulation in Mental Disorders: Current Evidence in the Field

**DOI:** 10.3389/fgene.2019.00580

**Published:** 2019-06-25

**Authors:** Błażej Misiak, Laura Ricceri, Maria M. Sąsiadek

**Affiliations:** ^1^Department of Genetics, Wrocław Medical University, Wrocław, Poland; ^2^Centre for Behavioural Sciences and Mental Health, Istituto Superiore di Sanità, Rome, Italy

**Keywords:** retrotransposon, DNA methylation, LINE-1, Alu, SINE, SVA

## Abstract

Transposable elements (TEs) are highly repetitive DNA sequences in the human genome that are the relics of previous retrotransposition events. Although the majority of TEs are transcriptionally inactive due to acquired mutations or epigenetic processes, around 8% of TEs exert transcriptional activity. It has been found that TEs contribute to somatic mosaicism that accounts for functional specification of various brain cells. Indeed, autonomous retrotransposition of long interspersed element-1 (LINE-1) sequences has been reported in the neural rat progenitor cells from the hippocampus, the human fetal brain and the human embryonic stem cells. Moreover, expression of TEs has been found to regulate immune-inflammatory responses, conditioning immunity against exogenous infections. Therefore, aberrant epigenetic regulation and expression of TEs emerged as a potential mechanism underlying the development of various mental disorders, including autism spectrum disorders (ASD), schizophrenia, bipolar disorder, major depression, and Alzheimer’s disease (AD). Consequently, some studies revealed that expression of some sequences of human endogenous retroviruses (HERVs) appears only in a certain group of patients with mental disorders (especially those with schizophrenia, bipolar disorder, and ASD) but not in healthy controls. In addition, it has been found that expression of HERVs might be related to subclinical inflammation observed in mental disorders. In this article, we provide an overview of detrimental effects of transposition on the brain development and immune mechanisms with relevance to mental disorders. We show that transposition is not the only mechanism, explaining the way TEs might shape the phenotype of mental disorders. Other mechanisms include the regulation of gene expression and the impact on genomic stability. Next, we review current evidence from studies investigating expression and epigenetic regulation of specific TEs in various mental disorders. Most consistently, these studies indicate altered expression of HERVs and methylation of LINE-1 sequences in patients with ASD, schizophrenia, and mood disorders. However, the contribution of TEs to the etiology of AD is poorly documented. Future studies should further investigate the mechanisms linking epigenetic processes, specific TEs and the phenotype of mental disorders to disentangle causal associations.

## Introduction

Mental disorders represent complex phenotypes and are the leading causes of global disease burden (Vigo et al., 2016). The phenotype complexity of mental disorders manifests in symptomatic and biological overlap, impeding a diagnostic process that is based on a clinical consensus without a crucial role of biological markers. Heritability rates of mental disorders are high, exceeding 80% in twin studies of schizophrenia and bipolar disorder (Cardno and Gottesman, 2000; McGuffin et al., 2003; Misiak et al., 2016). However, monogenic determinants with high penetrance rates have not been identified so far, and the concept of major mental disorders as the polygenic phenotypes prevails in the research approaches. Consequently, a paradigm shift toward investigating polygenic signatures, gene × environment (G × E) interactions and epigenetic mechanisms has been widely observed in the recent years.

The term ‘epigenetics’ refers to a number of reversible mechanisms that impacts gene expression without altering DNA sequence, and include DNA methylation and hydroxymethylation at the CpG islands, histone modifications as well as the regulation by microRNA species. It is now increasingly being recognized that the brain development is a complex process during which there is an increased sensitivity to the regulatory effects of epigenetic mechanisms (Nagy and Turecki, 2012). In light of existing evidence, major mental disorders, especially schizophrenia and autism spectrum disorders (ASD), are perceived as the neurodevelopmental disorders, occurring due to the effects of various genetic and environmental factors that affect critical periods of the brain development (Meredith, 2015).

Transposable elements (TEs) are the highly repetitive DNA sequences that constitute more than 50% of the human genome and contain about 52% of all CpG dinucleotides (Cordaux and Batzer, 2009; Su et al., 2012). Therefore, methylation at TEs is believed to serve as a proxy measure of global DNA methylation. Some TEs share similarity to exogenous viral agents and thus they are called endogenous retroviruses (Griffiths, 2001). Only about 7% of TEs are transcriptionally active (Oja et al., 2008). It has been estimated that approximately 0.27% of human genetic diseases are caused by retrotransposition (Callinan and Batzer, 2006).

Less is known about the contribution of TEs to the etiology of mental disorders. However, there is accumulating evidence that retrotransposition plays an important role in shaping somatic mosaicism that accounts for functional specification of brain cells (Baillie et al., 2011; Poduri et al., 2013). For instance, it has been reported that the transposition of long interspersed element (LINE)-1 sequences may play a role in differentiation of neurons during the brain development (Muotri et al., 2010). Moreover, this sequence exerts autonomous retrotransposition activity in the neural rat progenitor cells from the hippocampus, the human fetal brain and the human embryonic stem cells (Muotri et al., 2005; Coufal et al., 2009). Therefore, aberrant epigenetic regulation of TEs has been hypothesized to play an important role in the development of mental disorders. In this article, we provide an overview of transposition processes with relevance to major psychiatric disorders. Next, we review human and animal model studies investigating expression and epigenetic regulation of TEs in various mental disorders. Finally, we provide a summary of evidence with future directions and potential translation of findings to personalized precision medicine.

## Brief Overview of TEs in the Human Genome – Classification and Nomenclature

**FIGURE 1:**
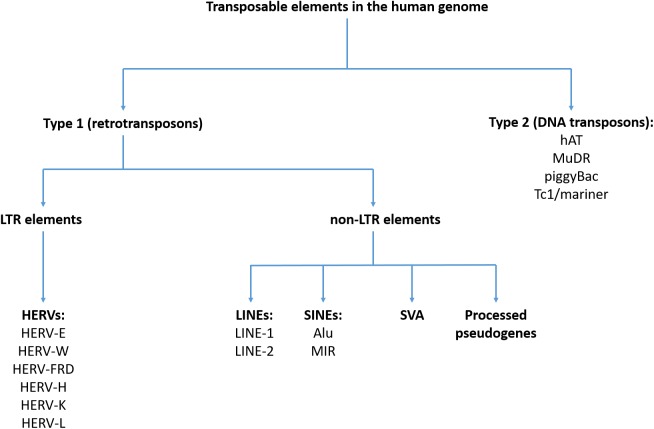
Classification of transposable elements in the human genome.

Classification of TEs in the human genome was shown in Figure 1. A detailed description of the structure and function of various TEs can be found elsewhere (Darby and Sabunciyan, 2014). All TEs can be divided into two subgroups – type I TEs (retrotransposons) and type II TEs (DNA transposons). Type I TEs can be divided into two subgroups – long terminal repeat (LTR) elements, represented by the human endogenous retroviruses (HERVs) and non-LTR sequences that include LINEs, short interspersed elements (SINEs) and processed pseudogenes (Dewannieux and Heidmann, 2005). Retrotransposons act via RNA intermediates that are converted to DNA sequences before transposition (reverse transcription) (Munoz-Lopez and Garcia-Perez, 2010). Type II TEs encode enzymes required for insertion and excision, enabling direct transposition processes without the use of RNA intermediates (Pray, 2008b). Some TEs are autonomous and encode all enzymes that are necessary for transposition, while the rest of them require a transcriptional activity of other transposons. Type II TEs have lost a transposition activity (Darby and Sabunciyan, 2014).

The HERV sequences have likely existed as exogenous infectious factors; however, they have lost this activity due to acquired mutations (Bannert and Kurth, 2006). These TEs constitute 8% of the human genome and contain genes that are conservative for all retroviruses, including the *gag, pro, pol*, and *env* genes (Lander et al., 2001; Vargiu et al., 2016). The *gag* gene encodes proteins that build up matrix, capsid and nucleocapsid. *Pro* and *pol* encode protease, reverse transcriptase and integrase. In turn, the *env* gene is expressed to surface and transmembrane proteins. The HERV sequences in the human genome represent three classes of retroviruses: class I (e.g., HERV-E, HERV-W, HERV-FRD, and HERV-H), class II (e.g., HERV-K), and class III (e.g., HERV-L). This classification is based on the similarity to exogenous retroviruses. The HERV-K sequences are the youngest and exert the highest transcriptional activity. The HERV sequences can provide promoters, enhancers, repressors, poly-A signals and alternative splicing sites for human transcripts (Vargiu et al., 2016).

The LINEs that represent non-LTR elements, possess an autonomous retrotransposition activity and include LINE-1 and LINE-2 sequences. These sequences make up approximately 21% of the human genome (Lander et al., 2001; Schumann et al., 2010). The LINE-1 sequences contain their own promoters and encode two open reading frame proteins – ORF1 that is an RNA-binding protein and ORF2 with endonuclease and reverse transcriptase activities. They are the most abundant sequences from the LINE family, making up 18% of the human genome (Lander et al., 2001). The majority of LINE-1 sequences are transcriptionally inactive. The LINE-2 sequences in the human genome are highly defective and can encode either one or two ORF proteins (Darby and Sabunciyan, 2014).

The SINEs are active and non-autonomous TEs, represented by the Alu and the Mammalian wide Interspersed Repeat (MIR) elements (11 and 3% of the human genome, respectively). The Alu sequences were named based on sharing a common cleavage site for the *Alu*I restriction enzyme (Houck et al., 1979). The Alu sequences are active but require the reverse transcriptase that is encoded by LINE-1 sequences (Pray, 2008a). In turn, the MIR elements are inactive. It has recently been shown that SINEs may form more complex sequences that are classified as the SVA retrotransposons. The SVA sequences have been formed by coupling the SINEs, a variable number of tandem repeats and the Alu retrotransposons. The SVAs also require the LINE-1 expression for mobilization. These sequences contribute to about 0.1% of the human genome and are the most active group of retrotransposons (Ostertag et al., 2003; Wang et al., 2005).

In turn, pseudogenes are DNA sequences that are related to real genes but they have lost at least some protein-coding abilities. It has been found that mRNA of pseudogenes can be reverse transcribed by the proteins encoded by LINE-1 sequences and transferred into other regions of the genome, creating processed pseudogenes. It has been estimated that the human genome consists of over 7,800 pseudogenes (Zhang et al., 2003). In case of integration close to active promoters, processed pseudogenes can be further transcribed. As listed by Kazazian (2014), they share the following characteristics: (1) their sequences are similar to the transcribed part of the parent gene; (2) they lack all or most introns; (3) they contain a poly-A tail attached to the 3′-most transcribed nucleotide; and (4) they are flanked at their 5′ and 3′ terminals by target site duplications of 5–20 nucleotides.

Finally, little is known about type II TEs (DNA transposons) that have never been active in the human genome. Type II TEs include the hAT, MuDR, piggyBac, and Tc1/mariner sequences (Munoz-Lopez and Garcia-Perez, 2010). These transposons do not act via RNA intermediates and encode enzymes that enable their mobilization. Due to their inactivity their causal role in the etiology of human diseases is less likely (Darby and Sabunciyan, 2014).

## Insights Into Potential Mechanisms Underlying the Role of TEs in Mental Disorders

A recent review of human monogenic diseases that occur due to retrotransposition suggests that only the transposition of LINE-1, Alu, and SVA sequences might be deleterious, underlying the development of monogenic diseases (Kaer and Speek, 2013). Retrotransposition might affect various gene regions via altering their sequence or influencing expression activity. For instance, the Alu sequences contain several stop codons that may result in a truncated protein (Mighell et al., 1997). This mechanism has been discovered in patients with hemophilia B caused by transferring the Alu-Ya5 element into a protein coding region of the factor IX gene (Vidaud et al., 1993). In case of transposition into promoter regions, these sequences might impact gene expression. Another scenario originates from sequence homology that can promote homologous recombination, leading to insertions and deletions. Finally, the SVA tandems can mobilize exons, contributing to complex rearrangements.

However, the effects of alterations in DNA sequence triggered by retrotransposition have not been found to underlie the development of common mental disorders. In the majority of studies of patients with mental disorders (reviewed in detail below), altered expression and/or epigenetic regulation of retrotransposons have been reported. There are several epigenetic processes that act as defense mechanism against retrotransposition, including DNA methylation, histone modifications, small RNA-mediated regulation and post-transcriptional silencing by DICER and siRNA (Lapp and Hunter, 2016). Indeed, the majority of TEs in the human genome are hypermethylated (Pray, 2008b). Although DNA methylation acts as a defense mechanism, it cannot be excluded that hypermethylation of newly inserted TEs can lead to further changes in chromatin conformation, triggering changes in the expression of adjacent genes. It is most likely that retrotransposition occurs during early development when epigenetic marks are removed (Darby and Sabunciyan, 2014). There are also some well characterized histone modifications, including trimethylation of lysine 9 and lysine 27 at histone H3 (H3K9me3 and H3K27me3, respectively), which lead to heterochromatin formation and transcriptional silencing of TEs (Day et al., 2010; Baker et al., 2012).

It should be noted that only a small subset of TEs has been reported to be involved in retrotransposition. For instance, only 30–60 LINE-1 sequences in diploid cells are capable of retrotransposition (Sassaman et al., 1997). In addition, the majority of LINE-1 sequences are methylated to a certain degree. It has been found that LINE-1 methylation might impact gene expression via specific mechanisms [for review see (Kitkumthorn and Mutirangura, 2011)]. Firstly, LINE-1 sequences may produce unique RNA transcripts that act beyond the LINE-1 location. Alternatively, the reverse LINE-1 promoter can transcribe unique DNA sequences beyond the 5′ end of LINE-1. The second scenario is that intragenic LINE-1 RNAs decrease the expression of host gene via the nuclear RNA-induced silencing complexes. Indeed, it has been found that the Argonaute-2 (AGO2) protein targets intronic LINE-1 pre-mRNA complexes leading to down-regulation of gene expression in cancer cells (Aporntewan et al., 2011).

Global DNA hypomethylation that progresses with aging has been associated with genomic instability (Jung and Pfeifer, 2015). Hypomethylated genome regions are prone to accumulate various types of DNA lesions that include oxidative damage, depurination, depyrimidation and pathologic endogenous double-strand breaks (Mutirangura, 2019). The latter ones are now believed to act as intermediate products that drive genomic instability (Mutirangura, 2019). Accumulating evidence indicates that methylation of TEs might protect against genomic instability processes. For instance, it has been demonstrated that Alu siRNA increases Alu methylation levels, lowers endogenous DNA damage and increases DNA resistance to DNA damaging agents (Patchsung et al., 2018). Similarly, LINE-1 hypomethylation may contribute to genomic instability via interactions with the *ATM* gene expression (Kitkumthorn and Mutirangura, 2011). However, the contribution of a reduction in the Alu methylation to genomic instability might be greater than that of LINE-1 or HERV sequences (Jintaridth and Mutirangura, 2010).

It remains largely unknown how changes in the expression of TEs might contribute to the development of mental disorders. It has been hypothesized that the presence of TEs in the human genome provides immunity against several infectious agents. Indeed, the mechanisms that contributed to HERV insertions are analogous to those used for replication by exogenous retroviruses (Grandi and Tramontano, 2018). Therefore, changes in the expression of TEs, e.g., via epigenetic processes, might impact immune responses and make the host more liable to exogenous infections. There is evidence that HERV-derived peptides may interact with innate immunity via various mechanisms. For instance, HERV proteins are able to interact with pattern recognition receptors (PRRs) that play a pivotal role in antiviral responses (Hurst and Magiorkinis, 2015). Emerging evidence indicates that exogenous viruses, including herpesviruses and influenza virus, might modulate the expression of HERV sequences. This mechanism might play a protective role and has been reviewed in detail by Grandi and Tramontano (2018). In brief, HERV transcripts might interact with homologous RNA from exogenous retroviruses, leading to the formation of molecules that are recognized by PRRs, acting as innate immunity sensors. The similarity of HERV proteins to those exogenous retroviruses allow them to compete with cellular receptors. This similarity might also trigger complementation events that impair formation of viral particles after cellular infection. On the other site, HERV proteins may suppress innate immunity. It has been reported that HERV-K proteins inhibit the activation of T cells (Morozov et al., 2013) as well as reduce the levels of interleukin-6 and Toll-like Receptor 7 (Laska et al., 2017).

## Transposable Elements and Their Epigenetic Regulation in Mental Disorders

As mentioned above, expression of TEs might play an important role in shaping immune responses against exogenous infections. Aberrant immune-inflammatory responses have been reported in several mental disorders. Also, a number of exogenous infections have been found to impact a risk of mental disorders. Below, we review studies investigating TEs and their epigenetic regulation in specific mental disorders, starting from the rationale of these studies that is based on the contribution of immune-inflammatory processes. A summary of human studies was provided in Table 1.

**Table 1:** Overview of human studies investigating the role of TEs in mental disorders.

Diagnosis	Study	*N*	Methods	Main findings
ASD	Balestrieri et al., 2012	28 ASD children 28 HCs	Expression of HERV sequences (E, H, K, and W) in PBMCs – RT-PCR	The percentage of HERV-H and HERV-W positive samples was significantly higher in ASD patients compared to HCs. HERV-H was more abundantly expressed and HERV-W had lower abundance in ASD patients than in HCs. PBMCs from ASD patients had an increased potential to up-regulate the HERV-H expression upon stimulation.
	Balestrieri et al., 2016	30 ASD children 30 HCs	Expression of HERV sequences (H, K, and W) in PBMCs – RT-PCR	There were significantly higher expression levels of HERV-H in PBMCs from ASD children compared to HCs. In turn, expression levels of HERV-K and HERV-W were significantly higher in PBMCs from healthy controls compared to ASD children.
	Saeliw et al., 2018	36 ASD patients 20 HCs Publicly available datasets of DNA expression profiling (465 ASD patients and 276 HCs)	Methylation (COBRA) and expression of Alu sequences (RT-PCR) in lymphoblastoid cell lines	A total of 320 Alu-inserted genes were differentially expressed. These genes are known to be associated with neurodevelopmental and neurological disorders. Pathway analysis revealed that these genes are involved in inflammation, estrogen receptor and androgen signaling.
	Shpyleva et al., 2018	13 ASD patients 13 HCs	LINE-1 methylation (the 5-methylcytosine MeDIP assay) and expression (RT-PCR) as well as H3K9me3 (ChIP) in post-mortem brain samples	LINE-1 expression was significantly higher in the cerebellum but not in the BA9, BA22, and BA24 brain regions from ASD patients. The binding of repressive MeCP2 protein and histone H3K9me3 to LINE-1 was significantly lower in the cerebellum of ASD patients. Higher LINE-1 expression was associated with elevated levels of oxidative stress. Overall, no significant differences in methylation levels between ASD patients and HCs were found. However, significantly altered Alu methylation patterns were observed in ASD cases sub-grouped based on clinical manifestation compared with HCs.
SCZ	Bundo et al., 2014	48 SCZ patients 13 BD patients 12 MDD patients 47 HCs	The number of LINE-1 copies in brain samples and induced pluripotent cells (RT-PCR)	The LINE-1 retrotransposition in neurons from the prefrontal cortex of patients with schizophrenia was increased, especially in the genes involved in synaptic functions. These findings were confirmed in induced pluripotent cells from patients with 22q11 deletion syndrome as well as in a mouse model of schizophrenia (maternal immune activation paradigm).
	Doyle et al., 2017	36 SCZ patients 26 HCs	LINE-1 insertions in the dorsolateral prefrontal cortex samples (qPCR)	A significant increase in the number of intragenic LINE-1 insertions has been observed in the dorsolateral prefrontal cortex of patients with schizophrenia compared to healthy controls.
	Fachim et al., 2019	35 FES patients 21 siblings of SCZ patients 35 HCs	LINE-1 methylation (pyrosequencing) in peripheral blood leukocytes	LINE-1 methylation was significantly higher in FES patients and siblings of schizophrenia patients compared to HCs.
	Frank et al., 2005	39 SCZ patients 39 HCs	Expression of HERV sequences in brain samples (HERV chip and RT-PCR)	Overrepresentation of the HERV-K(HML2) group in the brain samples of SCZ patients was found.
	Huang et al., 2006	58 recent-onset SCZ patients 38 HCs	Expression of the HERV *pol* gene in peripheral blood leukocytes (RT-PCR)	The HERV pol gene expression was detected in 34.5% of SCZ patients but not in HCs.
	Kalayasiri et al., 2019	31 paranoid SCZ patients 94 MIP patients 163 HCs	LINE-1 methylation in PBMCs (COBRA)	Methylation levels of LINE-1 sequences were significantly higher in paranoid SCZ patients and MIP patients compared to HCs.
	Karlsson et al., 2002	35 FES and FESaff patients 20 SCZ and SCZaff patients 22 patients with non-inflammatory neurological disorders 30 HCs	Detection of HERV sequences in CSF (cDNA synthesis, pan-retroviral PCR, cloning and sequencing)	Nucleotide sequences homologous to those of the HERV-W *pol* gene were found in CSF of 28.6% of FES patients and in 5% of patients with SCZ and SCZaff. These sequences were not detected in the CSF of individuals with non-inflammatory neurological diseases and healthy controls.
	Karlsson et al., 2004	54 FES and FESaff patients 46 HCs	The presence of HERV-W sequences in plasma samples (RT-PCR)	The HERV-W gag sequences were detected in 16.7% of patients and 4.3% of HCs.
	Li et al., 2019	32 SCZ patients 51 HCs	Analysis of post-mortem brain samples (RNAseq data)	Increased transcription of HERV, especially HERV-W and HERV-H elements, was found in the anterior cingulate cortex, hippocampus, and orbitofrontal cortex of patients with SCZ.
	Mak et al., 2019	100 MES patients 49 FES patients 97 HCs	Methylation of HERV-K sequences in peripheral blood leukocytes (COBRA)	There were no significant differences in the level of HERV-K methylation between MES patients and HCs. In turn, FES patients had significantly lower HERV-K methylation than HCs. There was a significant positive correlation between a cumulative dosage of antipsychotic and the HERV-K methylation level in MES patients.
	Melbourne et al., 2018	17 SCZ patients 16 HCs	Expression of HERV-W *env* and *gag* in PBMCs (RT-PCR)	There were no significant differences in the level of expression. In all participants, higher expression of HERV-W *env* and *gag* was related to higher levels of interleukin-6. There was a negative correlation between the dosage of atypical antipsychotic and the level of HERV-W *env* and *gag* expression.
	Misiak et al., 2015	48 FES patients 48 HCs	Methylation of LINE-1 sequences in peripheral blood leukocytes (COBRA)	There were no significant differences in the level of LINE-1 methylation between FES patients and HCs. However, FES patients with a positive history of childhood trauma had significantly lower LINE-1 methylation than HCs. More specifically, a higher level of emotional abuse was related to lower LINE-1 methylation in FES patients.
	Perron et al., 2012	45 SCZ patients 91 BD patients 73 HCs	Expression of HERV-W env gene in PBMCs (RT-PCR)	There were significantly elevated transcription levels of the HERV-W *env* sequence in SCZ patients compared to HCs. However, the levels of HERV-W *env* expression were significantly lower compared to BD patients.
	Weis et al., 2007	15 SCZ patients 15 HCs	Expression of HERV-W gag protein in brain samples (immunohistochemistry)	Expression level of the HERV-W gag protein has been found to be decreased in the cingulate gyrus and the hippocampus of patients with SCZ.
Mood disorders	Frank et al., 2005	38 BD patients 39 HCs	Expression of HERV sequences in brain samples (HERV chip and RT-PCR)	Overrepresentation of the HERV-K(HML2) group in the brain samples of BD patients was found.
	Li et al., 2018	24 BD patients 51 HCs	Analysis of post-mortem brain samples (RNAseq data)	Increased transcription of HERV, especially HERV-W and HERV-H elements, was found in the anterior cingulate cortex, hippocampus, and orbitofrontal cortex of patients with BD.
	Perron et al., 2012	91 BD patients 45 SCZ patients 73 HCs	Expression of HERV-W env gene in PBMCs (RT-PCR)	There were elevated transcription levels of the HERV-W env sequence in SCZ patients compared to SCZ and HCs.
	Weis et al., 2007	15 BD patients 15 MDD patients 15 HCs	Expression of HERV-W gag protein in brain samples (immunohistochemistry)	Expression level of the HERV-W gag protein has been found to be decreased in the cingulate gyrus and the hippocampus of patients with BD and SCZ.
AD	Bollati et al., 2011	43 AD patients 38 HCs	Methylation of LINE-1 sequences in peripheral blood leukocytes (pyrosequencing)	Increased LINE-1 methylation level was found in AD patients, especially those with better cognitive performance.
	Hernández et al., 2014	28 AD patients 30 HCs	Methylation of LINE-‘ sequences in peripheral blood leukocytes (MS-HRM)	No significant differences in LINE-1 methylation levels between AD patients and HCs were found.
	Protasova et al., 2017	18 AD patients 20 HCs	LINE-1 – the number of copies and expression level in post-mortem samples of frontal cortex (RT-PCR)	No significant differences in LINE-1 expression or the number of LINE-1 copies between AD patients and HCs were found.


*AD, Alzheimer’s disease; ASD, autism-spectrum disorder; BD, bipolar disorder; COBRA, combined bisulfite restriction analysis; CSF, cerebrospinal fluid; FES, first-episode schizophrenia; FESaff, first-episode schizoaffective disorder; H3K9me3, histone H3 lysine 9 trimethylation; HCs, healthy controls; MeCP2, methyl CpG binding protein 2; MDD, major depressive disorder; MIP, methamphetamine-induced psychosis; MS-HRM, methylation-sensitive high-resolution melting; PBMCs, peripheral blood mononuclear cells; RT-PCR, real-time polymerase chain reaction; SCZ, schizophrenia; SCZaff, schizoaffective disorder; qPCR, quantitative polymerase chain reaction*.

### Autism Spectrum Disorders (ASD)

Overexpression of HERV-H has been observed in peripheral blood mononuclear cells (PBMC) of children with ASD (Balestrieri et al., 2012, 2016). Similar findings have also been observed in two different ASD mouse models – inbred BTBR T+tf/J mice and CD-1 outbred mice prenatally exposed to valproic acid. In both of these mouse models, the expression of several endogenous retrovirus (ERV) families (ETnI, ETnII-α, ETnII-β, ETnII-γ, MusD, and IAP) was significantly higher in comparison with corresponding controls (Cipriani et al., 2018). Interestingly, the studies in mouse models provide additional information on the potential use of ERV sequences as biomarkers: (i) a higher expression of ERV was observed both in the peripheral blood mononuclear cells and the brain, suggesting that altered profile of peripheral ERV sequences may reflect similar alterations at the brain level; (ii) ERV overexpression in ASD mouse models is detectable from prenatal stage till the adulthood and (iii) ERV overexpression in ASD mouse models is also accompanied by increased expression of pro-inflammatory cytokines and Toll-like receptors. Furthermore, a subsequent study in one of the models (mice prenatally exposed to valproic acid) provided evidence that higher levels of ERVs are also detectable in the offspring (second and third generations) of those mice exposed prenatally to valproic acid (Tartaglione et al., 2018).

Also LINE-1 retrotransposons have been associated with ASD (Shpyleva et al., 2018; Suarez et al., 2018). The levels of LINE-1 ORF1 and ORF2 transcripts have been investigated in four brain regions of patients with idiopathic autism (the frontal cortex, anterior cingulate, auditory cortex, and cerebellum). Elevated LINE-1 expression together with lower binding affinity of repressive MeCP2 protein and histone H3K9me3 to LINE-1 sequences was observed only in the cerebellum, suggesting a lessening of epigenetic repression and consequently an increase in chromatin accessibility. Interestingly, the increase in LINE-1 expression was also inversely correlated with glutathione redox status, consistent with reports indicating that LINE-1 expression is increased under pro-oxidant conditions (Shpyleva et al., 2018). The overexpression of LINE-1 within a single brain region is suggestive of a mosaicism-like impact of retrotransposons and definitively needs further investigation. In partial agreement with the findings of increased LINE-1 expression in ASD, data concerning LINE-1 methylation status in lymphoblastoid peripheral cells have provided evidence of reduced methylation in a subgroup of patients with severe language impairment (Tangsuwansri et al., 2018).

It has also been shown that the Alu sequence, the most abundant of all TEs in the human genome, deserves further research in ASD (Saeliw et al., 2018). Indeed, this study investigated the Alu methylation and expression in lymphoblastoid peripheral cells from ASD patients. Although the global methylation of Alu subfamilies was not significantly different between ASD and control group, when ASD samples were divided according to phenotypic subgroups, methylation patterns of the AluS subfamily were different from those in relative controls in two of the ASD subgroups, and within one of the subgroup (mild phenotype), the Alu expression was correlated with methylation status. Despite the limited sample size (particularly of subgroups), these data suggest that classification of ASD patients in phenotypic subgroups may represent a useful tool in investigating associations of TEs with the highly heterogeneous ASD diagnostic construct.

### Schizophrenia-Spectrum Disorders

It has been clearly demonstrated that winter-spring seasonality of birth as well as prenatal and postnatal infections increase a risk of developing schizophrenia (McGrath and Welham, 1999; Davies et al., 2003; Khandaker et al., 2013). Moreover, the largest genome-wide association study revealed that variation within the HLA genes is strongly associated with schizophrenia susceptibility (Ripke et al., 2014). Finally, schizophrenia patients present with several indices of subclinical inflammation in terms of pro-inflammatory cytokine profiles (Miller et al., 2011; Frydecka et al., 2018), alterations of lymphocyte counts (Miller et al., 2013; Karpiǹski et al., 2016, 2018) and elevated levels of C-reactive protein (CRP) (Fernandes et al., 2016). On the basis of a meta-analysis, Arias et al. (2012) found a higher prevalence of infections with several agents, including *Human Herpesvirus* (HHV) *2, Borna Disease Virus, Chlamydia pneumoniae, Chlamydia psittaci*, and *Toxoplasma gondii* in patients with schizophrenia compared to healthy controls.

Accumulating evidence indicates altered expression of HERV sequences in patients with schizophrenia. Karlsson et al. (2002) found nucleotide sequences homologous to those of the HERV-W *pol* gene in the cerebrospinal fluid (CSF) of 28.6% of first-episode schizophrenia patients and in 5% of patients with chronic schizophrenia. These sequences were not detected in the CSF of individuals with non-inflammatory neurological diseases and healthy controls. Increased levels of HERV-W-related *gag* and *pol* transcripts and a higher prevalence of the *gag* and *pol* antigenemia in peripheral blood from patients with schizophrenia compared to healthy controls have been reported by several studies (Karlsson et al., 2004; Huang et al., 2006; Perron et al., 2008; Yao et al., 2008). The study by Perron et al. (2008) also revealed significantly higher rates of positive HERV-W *env* antigenemia in patients with schizophrenia than in healthy controls. The HERV-W *gag* and *env* antigenemia has been also associated with subclinical inflammation in terms of elevated levels of CRP and pro-inflammatory cytokines (Perron et al., 2008; Melbourne et al., 2018). Interestingly, Huang et al. (2011) found that overexpression of the HERV-W *env* in the human U251 glioma cells up-regulated a number of schizophrenia-associated genes, including those that encode brain-derived neurotrophic factor, neurotrophic tyrosine kinase receptor type 2 and the dopamine D3 receptor as well as increased the phosphorylation of cyclic adenosine monophosphate response element-binding protein. In this study, mRNA of the HERV-W *env* gene was detected in plasma from 42 out of 118 recent-onset schizophrenia patients but not in healthy controls. There is also evidence that the expression of HERV-W *env* induces calcium influx and down-regulates the *DISC1* gene expression in the human neuroblastoma cells (Chen et al., 2018). Interestingly, expression level of the HERV-W *gag* protein has been found to be decreased in the cingulate gyrus and the hippocampus of patients with schizophrenia (Weis et al., 2007). However, a recent analysis of RNA-seq data in the human *post mortem* brain samples revealed increased transcription of HERV, especially HERV-W and HERV-H elements, in the anterior cingulate cortex, hippocampus and orbitofrontal cortex of patients with schizophrenia and bipolar disorder (Li et al., 2019). Interestingly, the HERV sequences within the *ERVWE1* gene (7q21.2) exhibited the highest levels of transcription across all brain regions examined in this analysis. The *env* gene in this locus encodes syncytin-1, expressed at high levels in the human placenta (Blond et al., 2000; Mi et al., 2000). However, altered expression of this gene has been reported in the areas of active demyelination in patients with multiple sclerosis (Mameli et al., 2007). At this point, it should be noted that myelin alterations are widely observed in patients with schizophrenia (Mighdoll et al., 2015). Although initial results regarding expression of the HERV-W sequences in schizophrenia patients are promising, caution should be taken on the way these results are being interpreted. Indeed, the majority of studies in this field analyzed the overall expression of HERV-W sequences without investigating specific HERV-W loci. Moreover, no conclusive association between the HERV-W expression and other human pathologies has been documented so far [for review see (Grandi and Tramontano, 2017)].

Less is known about other families of HERVs in patients with schizophrenia. Frank et al. (2005) found overrepresentation of the HERV-K(HML2) group in brain samples of patients with schizophrenia and bipolar disorder. Our group also tested peripheral blood methylation levels of HERV-K sequences in first-episode and multi-episode schizophrenia patients (Mak et al., 2019). We found significantly lower levels of HERV-K methylation in first-episode schizophrenia patients compared to healthy controls. These alterations were not observed in multi-episode schizophrenia patients. Moreover, we did not find an association between HERV-K methylation levels and the deficit schizophrenia subtype that refers to a subgroup of patients with enduring and persistent negative symptoms. However, we found a significant positive correlation between the dosage of antipsychotics and HERV-K methylation levels in multi-episode schizophrenia patients. These findings imply that the HERV-K methylation might normalize in the course of schizophrenia. It is also likely that antipsychotic drugs might impact methylation and expression of HERV-K sequences. In contrast to our findings, Diem et al. (2012) found no significant effects of valproic acid, haloperidol, risperidone and clozapine on the HERV-K expression levels in the human brain cell lines. However, valproic acid was found to strongly up-regulate expression of HERV-W and ERV9 elements.

Some studies also investigated methylation status and expression levels of non-LTR sequences in patients with schizophrenia. Bundo et al. (2014) demonstrated increased LINE-1 retrotransposition in neurons from the prefrontal cortex of patients with schizophrenia, especially in the genes involved in synaptic functions. These findings were confirmed in induced pluripotent cells from patients with 22q11 deletion syndrome as well as in a mouse model of schizophrenia (maternal immune activation paradigm). In agreement with these results, a significant increase in the number of intragenic LINE-1 insertions has been observed in the dorsolateral prefrontal cortex of patients with schizophrenia compared to healthy controls (Doyle et al., 2017). Over-representation of these insertions appeared within the gene ontologies called “cell projection” and “postsynaptic membrane,” suggesting their role in the brain development. In some studies, LINE-1 methylation was tested in peripheral blood leukocytes of patients with schizophrenia, providing mixed findings (Misiak et al., 2015; Li et al., 2018; Fachim et al., 2019; Kalayasiri et al., 2019). The study by our group revealed lower LINE-1 methylation only in patients with first-episode schizophrenia and a positive history of childhood trauma. Among various childhood adversities, emotional trauma was most strongly associated with the LINE-1 methylation status. These results are in agreement with a previous study, showing that the LINE-1 methylation might be involved in resilience and susceptibility to develop post-traumatic stress disorder (Rusiecki et al., 2012). Moreover, increased expression of LINE-1 in response to stress has been reported in various cell lines (Li and Schmid, 2001; Capomaccio et al., 2010). Lower LINE-1 methylation levels in patients with schizophrenia and bipolar disorder were also reported by Li et al. (2018). Other studies revealed hypermethylation of LINE-1 sequences in patients with first-episode psychosis, paranoid schizophrenia and methamphetamine-induced paranoia (Fachim et al., 2019; Kalayasiri et al., 2019).

### Mood Disorders

A recent systematic review indicates that prenatal infections might impact the risk of bipolar disorder (Marangoni et al., 2016). However, this observation is based on a lower number of studies compared to studies addressing the impact of prenatal infections on schizophrenia risk. There is evidence that influenza infection during pregnancy is associated with a fourfold increase in the risk of bipolar disorder in the offspring (Parboosing et al., 2013). Another study demonstrated that prenatal flu exposure increases the risk of bipolar disorder with psychotic features (Canetta et al., 2014). However, no association was found between prenatal infections with HHV-1, HHV-2, *Cytomegalovirus* or *Toxoplasma gondii* and bipolar disorder risk (Mortensen et al., 2011). Maternal infections in the second trimester might also contribute to the development of depressive symptoms in the adolescent offspring (Murphy et al., 2017). However, the impact of specific infectious agents has not been tested so far.

Although all major mental disorders are characterized by co-existing subclinical inflammation, some differences, regarding specific pro-inflammatory markers can be indicated (Goldsmith et al., 2016; Misiak et al., 2019). Therefore, it might be hypothesized that the mechanisms leading to subclinical inflammation in bipolar disorder, major depression and schizophrenia-spectrum disorders are different. However, studies investigating expression of TEs do not support this hypothesis. For instance, over-expression of HERV-K sequences has been reported in brain samples of patients with bipolar disorder and schizophrenia (Frank et al., 2005). Similarly, decreased expression of the HERV-W *gag* protein has been reported in the cingulate gyrus and hippocampus of patients with schizophrenia, bipolar disorder, and major depression (Weis et al., 2007). Finally, hypomethylation of LINE-1 elements in peripheral blood has been observed in patients with bipolar disorder and schizophrenia (Li et al., 2018). Some differences have been detected with respect to the expression of HERV-W sequences. Indeed, Perron et al. (2012) found elevated transcription levels of the HERV-W *env* sequence in the peripheral blood of patients with bipolar disorder and schizophrenia compared to healthy controls. Expression levels of the HERV-W *env* sequence were also significantly higher in patients with bipolar disorder than in those with schizophrenia.

### Alzheimer’s Disease

There is a general consensus that aging processes are associated with progressive loss of global DNA methylation and site-specific DNA hypermethylation (Jung and Pfeifer, 2015). Similarly, TEs are subjected to profound epigenetic modifications during aging that appear in the context of organismal and cellular senescence (Cardelli, 2018). For instance, age-related loss of Alu and HERV-K methylation has been well-documented (Bollati et al., 2009; Jintaridth and Mutirangura, 2010; Gentilini et al., 2013). Moreover, it has been found that the expression of HERV-H, HERV-K and HERV-W changes during the lifespan with distinct patterns (Balestrieri et al., 2015). Importantly, the study by Gentilini et al. (2013) demonstrated that age-related loss of Alu methylation was less apparent in the offspring of centenarians, suggesting the effects of genetic factors associated with longevity. In turn, studies investigating changes of LINE-1 methylation have provided mixed findings (Bollati et al., 2009; Talens et al., 2012; Cho et al., 2015). Finally, there is evidence that chromatin of Alu, SVA, and LINE-1 becomes relatively more open in senescent cells (De Cecco et al., 2013).

Age-related changes in epigenetic modifications of TEs have provided basis for investigating alterations of these processes in Alzheimer’s disease. In a single study of early-onset Alzheimer’s disease family, it has been reported that large genomic rearrangements might affect the presenilin-1 gene via the mechanisms involving recombination stimulated by the Alu sequence (Hiltunen et al., 2000). However, subsequent studies have not provided compelling evidence regarding the contribution of TEs to the etiology of Alzheimer’s disease. There is only one study, showing increased LINE-1 methylation in peripheral blood leukocytes of patients with Alzheimer’s disease, especially those with better cognitive performance, compared to healthy controls (Bollati et al., 2011). However, the authors did not find significant between-group differences in the levels of Alu and SAT-α methylation. Other studies did not confirm these findings regarding LINE-1 methylation (Hernández et al., 2014; Protasova et al., 2017). Alterations of other TEs in patients with Alzheimer’s disease have not been tested so far.

## Summary of Evidence and Future Directions

Although specific retrotransposition events that may account for mental disorders in the manner observed in case of Mendelian diseases have not been identified so far, accumulating evidence indicates the involvement of altered expression and epigenetic regulation of TEs in the pathophysiology of schizophrenia, mood disorders and ASD. Most consistently, previous studies indicate altered expression of HERVs and methylation of LINE-1 sequences. However, specific findings are similar in patients with various mental disorders and thus their use as biomarkers is largely limited. Moreover, the direction of causality is yet to be determined. For instance, it cannot be excluded that altered expression of HERV appears as a consequence of other epigenetic dysregulations that are widely observed in mental disorders. Additionally, severe mental disorders, including schizophrenia and mood disorders, are associated with high prevalence rates of somatic comorbidities, including autoimmune diseases, type 2 diabetes and cardiovascular diseases that have also been associated with altered epigenetic regulation of TEs (Cash et al., 2011; De Hert et al., 2011; Misiak et al., 2013; Nestler et al., 2016; Zhao et al., 2018). Interestingly, there are studies showing that the expression of various HERV sequences appears in a certain subgroup of patients with schizophrenia but not in healthy controls. These findings are consistent with previous studies, showing that immune alterations can be observed only in a subgroup of patients characterized by poor response to treatment and support the concept of psychosis subtypes (Frydecka et al., 2015; Mondelli et al., 2015; Fillman et al., 2016). Other clinical correlates of subclinical inflammation in schizophrenia include, i.e., more severe cognitive deficits (Misiak et al., 2017b), persistent negative symptoms (Goldsmith et al., 2018) and certain neurostructural abnormalities (Najjar and Pearlman, 2015). However, so far studies investigating expression and epigenetic regulation of TEs in schizophrenia have been based on relatively small samples without comprehensive clinical assessment. Similarly, studies investigating the expression of TEs in patients with bipolar disorder did not control for mood status and a severity of psychopathological symptoms.

Another important point is that causal inferences between TEs and mental disorders cannot be established. Firstly, it remains unknown what are the critical periods when alterations in epigenetic regulation and expression of TEs appear. Therefore, future studies should examine epigenetic processes that regulate expression of TEs in patients at early stages of mental disorders or individuals from clinical high risk groups. This is particularly important since several lifestyle characteristics that are highly prevalent among patients with mental disorders, e.g., cigarette smoking and poor dietary habits, might impact TEs *per se* (Miglino et al., 2014; Miousse et al., 2015). Secondly, the role of HERVs in shaping innate immunity also remains problematic with respect to understanding causal associations. On one side, expression of HERVs might condition resistance to exogenous infections; on the other, exogenous retroviruses have been found to impact the expression of HERVs. Therefore, it remains unknown whether altered expression profiles of HERVs in mental disorders represent cause or consequence of exogenous infections. Future studies should necessarily examine the biological nature and the extent of associations between immune alterations in mental disorders and expression of various TEs.

Finally, more global concordance patterns of different TEs expression in mental disorders are yet to be examined: this could provide further insight into specificity of methylation patterns across different TEs and provide additional information of their use as potential biomarkers. At this point, it is important to note that similar DNA methylation patterns have been described in brain samples and peripheral blood leukocytes of patients with schizophrenia (Van Den Oord et al., 2016).

Another direction for the field is to disentangle the effects of stressful life events on epigenetic regulation of TEs expression. Early-life stress is a known risk factor for mood and psychotic disorders as well as correlates with a number of biological dysregulations in adults (Misiak et al., 2017a; Bielawski et al., 2019; Jaworska-Andryszewska and Rybakowski, 2019). Acute stress has been found to increase the levels of H3K9me3 as well as decrease the levels of H3K9me1 and H3K27me3 in the dentate gyrus and the CA1 layer of the hippocampus in rats (Milne et al., 2009). In turn, chronic restraint stress for 21 days mildly increased the levels of H3Kme4 and reduced the levels of H3K9me3 in the dentate gyrus. Treatment with fluoxetine reversed changes in the levels of H3K9me3 during chronic restraint stress. More specifically, the same group found that acute stress had increased H3K9me3 enrichment at SINEs (Baker et al., 2012). In turn, our group found lower methylation of LINE-1 sequences in peripheral blood leukocytes of patients with first-episode schizophrenia reporting a positive history of childhood trauma (Misiak et al., 2015). In light of these findings, future studies should further examine the effects of stress on the expression of TEs in patients from various clinical groups and preclinical studies could contribute to this aim.

## Author Contributions

BM and MS conceived the concept of this article. BM wrote the Sections “Introduction”, “Insights Into Potential Mechanisms Underlying the Role of TEs in Mental Disorders”, “Schizophrenia-Spectrum Disorders” and “Mood Disorders”. LR wrote the Section “Autism Spectrum Disorders (ASD)”. MS prepared the Sections “Brief Overview of TEs in the Human Genome – Classification and Nomenclature” and “Alzheimer’s Disease”. BM, LR, and MS prepared the Section “Summary of Evidence and Future Directions”. All authors contributed to the manuscript revision, read, and approved the submitted version.

## Conflict of Interest Statement

The authors declare that the research was conducted in the absence of any commercial or financial relationships that could be construed as a potential conflict of interest.

## References

[B1] Aporntewan C. Phokaew C. Piriyapongsa J. Ngamphiw C. Ittiwut C. Tongsima S. (2011). Hypomethylation of intragenic LINE-1 represses transcription in cancer cells through AGO2. *PLoS One* 6:e17934. 10.1371/journal.pone.0017934 21423624 PMC3057998

[B2] Arias I. Sorlozano A. Villegas E. Luna J. de D. McKenney K. (2012). Infectious agents associated with schizophrenia: a meta-analysis. *Schizophr. Res.* 136 128–136. 10.1016/j.schres.2011.10.026 22104141

[B3] Baillie J. K. Barnett M. W. Upton K. R. Gerhardt D. J. Richmond T. A. De Sapio F. (2011). Somatic retrotransposition alters the genetic landscape of the human brain. *Nature* 479 534–537. 10.1038/nature10531 22037309 PMC3224101

[B4] Baker M. E. R. Pfaff D. W. McEwen B. S. Seligsohn M. Hunter R. G. Datson N. A. (2012). Acute stress and hippocampal histone H3 lysine 9 trimethylation, a retrotransposon silencing response. *Proc. Natl. Acad. Sci. U.S.A.* 109 17657–17662. 10.1073/pnas.1215810109 23043114 PMC3491472

[B5] Balestrieri E. Arpino C. Matteucci C. Sorrentino R. Pica F. Alessandrelli R. (2012). HERVs expression in autism spectrum disorders. *PLoS One* 7:e48831. 10.1371/journal.pone.0048831 23155411 PMC3498248

[B6] Balestrieri E. Cipriani C. Matteucci C. Capodicasa N. Pilika A. Korea I. (2016). Transcriptional activity of human endogenous retrovirus in Albanian children with autism spectrum disorders. *New Microbiol.* 39 228–231. 27704145

[B7] Balestrieri E. Pica F. Matteucci C. Zenobi R. Sorrentino R. Argaw-Denboba A. (2015). Transcriptional activity of human endogenous retroviruses in human peripheral blood mononuclear cells. *Biomed Res. Int.* 2015:164529. 10.1155/2015/164529 25734056 PMC4334862

[B8] Bannert N. Kurth R. (2006). The evolutionary dynamics of human endogenous retroviral families. *Annu. Rev. Genomics Hum. Genet.* 7 149–173. 10.1146/annurev.genom.7.080505.115700 16722807

[B9] Bielawski T. Misiak B. Moustafa A. Frydecka D. (2019). Epigenetic mechanisms, trauma, and psychopathology: targeting chromatin remodeling complexes. *Rev. Neurosci.* 10.1515/revneuro-2018-0055 [Epub ahead of print]. 30730846

[B10] Blond J. L. Lavillette D. Cheynet V. Bouton O. Oriol G. Chapel-Fernandes S. (2000). An envelope glycoprotein of the human endogenous retrovirus HERV-W is expressed in the human placenta and fuses cells expressing the type D mammalian retrovirus receptor. *J. Virol.* 74 3321–3329. 10.1128/jvi.74.7.3321-3329.2000 10708449 PMC111833

[B11] Bollati V. Galimberti D. Pergoli L. Dalla Valle E. Barretta F. Cortini F. (2011). DNA methylation in repetitive elements and Alzheimer disease. *Brain Behav. Immun.* 25 1078–1083. 10.1016/j.bbi.2011.01.017 21296655 PMC3742099

[B12] Bollati V. Schwartz J. Wright R. Litonjua A. Tarantini L. Suh H. (2009). Decline in genomic DNA methylation through aging in a cohort of elderly subjects. *Mech. Ageing Dev.* 130 234–239. 10.1016/j.mad.2008.12.003 19150625 PMC2956267

[B13] Bundo M. Toyoshima M. Okada Y. Akamatsu W. Ueda J. Nemoto-Miyauchi T. (2014). Increased L1 retrotransposition in the neuronal genome in schizophrenia. *Neuron* 81 306–313. 10.1016/j.neuron.2013.10.053 24389010

[B14] Callinan P. A. Batzer M. A. (2006). Retrotransposable elements and human disease. *Genome Dis.* 1 104–115. 10.1159/000092503 18724056

[B15] Canetta S. E. Bao Y. Co M. D. T. Ennis F. A. Cruz J. Terajima M. (2014). Serological documentation of maternal influenza exposure and bipolar disorder in adult offspring. *Am. J. Psychiatry* 171 557–563. 10.1176/appi.ajp.2013.13070943 24480930 PMC4025955

[B16] Capomaccio S. Verini-Supplizi A. Galla G. Vitulo N. Barcaccia G. Felicetti M. (2010). Transcription of LINE-derived sequences in exercise-induced stress in horses. *Anim. Genet.* 41(Suppl. 2), 23–27. 10.1111/j.1365-2052.2010.02094.x 21070272

[B17] Cardelli M. (2018). The epigenetic alterations of endogenous retroelements in aging. *Mech. Ageing Dev.* 174 30–46. 10.1016/j.mad.2018.02.002 29458070

[B18] Cardno A. G. Gottesman I. I. (2000). Twin studies of schizophrenia: from bow-and-arrow concordances to star wars Mx and functional genomics. *Am. J. Med. Genet. Semin. Med. Genet.* 97 12–17. 10.1002/(sici)1096-8628(200021)97:1<12::aid-ajmg3>3.3.co;2-l 10813800

[B19] Cash H. L. Mcgarvey S. T. Houseman E. A. Marsit C. J. Hawley N. L. Lambert-Messerlian G. M. (2011). Cardiovascular disease risk factors and DNA methylation at the LINE-1 repeat region in peripheral blood from Samoan Islanders. *Epigenetics* 6 1257–1264. 10.4161/epi.6.10.17728 21937883 PMC3225843

[B20] Chen Y. Yan Q. Zhou P. Li S. Zhu F. (2018). HERV-W env regulates calcium influx via activating TRPC3 channel together with depressing DISC1 in human neuroblastoma cells. *J. Neurovirol.* 25 101–113. 10.1007/s13365-018-0692-7 30397826

[B21] Cho Y. H. Woo H. D. Jang Y. Porter V. Christensen S. Hamilton R. F. (2015). The association of LINE-1 hypomethylation with age and centromere positive micronuclei in human lymphocytes. *PLoS One* 10:e0133909. 10.1371/journal.pone.0133909 26196382 PMC4510364

[B22] Cipriani C. Ricceri L. Matteucci C. De Felice A. Tartaglione A. M. Argaw-Denboba A. (2018). High expression of Endogenous Retroviruses from intrauterine life to adulthood in two mouse models of Autism Spectrum Disorders. *Sci. Rep.* 8:629. 10.1038/s41598-017-19035-w 29330412 PMC5766538

[B23] Cordaux R. Batzer M. A. (2009). The impact of retrotransposons on human genome evolution. *Nat. Rev. Genet.* 10 691–703. 10.1038/nrg2640 19763152 PMC2884099

[B24] Coufal N. G. Garcia-perez J. L. Peng G. E. Yeo G. W. Mu Y. Lovci M. T. (2009). L1 retrotransposition in human neural progenitor cells Nicole. *Nature* 460 1127–1131. 10.1038/nature08248.L1 19657334 PMC2909034

[B25] Darby M. M. Sabunciyan S. (2014). Repetitive elements and epigenetic marks in behavior and psychiatric disease. *Adv. Genet.* 86 185–252. 10.1016/B978-0-12-800222-3.00009-7 25172351

[B26] Davies G. Welham J. Chant D. Torrey E. F. McGrath J. (2003). A systematic review and meta-analysis of Northern Hemisphere season of birth studies in schizophrenia. *Schizophr. Bull.* 29 587–593. 10.1093/oxfordjournals.schbul.a007030 14609251

[B27] Day D. S. Luquette L. J. Park P. J. Kharchenko P. V. (2010). Estimating enrichment of repetitive elements from high-throughput sequence data. *Genome Biol.* 11:R69. 10.1186/gb-2010-11-6-r69 20584328 PMC2911117

[B28] De Cecco M. Criscione S. W. Peckham E. J. Hillenmeyer S. Hamm E. A. Manivannan J. (2013). Genomes of replicatively senescent cells undergo global epigenetic changes leading to gene silencing and activation of transposable elements. *Aging Cell* 12 247–256. 10.1111/acel.12047 23360310 PMC3618682

[B29] De Hert M. Correll C. U. Bobes J. Cetkovich-Bakmas M. Cohen D. A. N. Asai I. (2011). Physical illness in patients with severe mental disorders. I. Prevalence, impact of medications and disparities in health care. *World Psychiatry* 10 52–77. 10.1002/j.2051-5545.2011.tb00014.x 21379357 PMC3048500

[B30] Dewannieux M. Heidmann T. (2005). LINEs, SINEs and processed pseudogenes: parasitic strategies for genome modeling. *Cytogenet. Genome Res.* 110 35–48. 10.1159/000084936 16093656

[B31] Diem O. Schäffner M. Seifarth W. Leib-Mösch C. (2012). Influence of antipsychotic drugs on human endogenous retrovirus (HERV) transcription in brain cells. *PLoS One* 7:e30054. 10.1371/journal.pone.0030054 22253875 PMC3256206

[B32] Doyle G. A. Crist R. C. Karatas E. T. Hammond M. J. Ewing A. D. Ferraro T. N. (2017). Analysis of LINE-1 elements in DNA from postmortem brains of individuals with schizophrenia. *Neuropsychopharmacology* 42 2602–2611. 10.1038/npp.2017.115 28585566 PMC5686486

[B33] Fachim H. Loureiro C. Corsi-Zuelli F. Shuhama R. Louzada-Junior P. Menezes P. (2019). GRIN2B promoter methylation deficits in early-onset schizophrenia and its association with cognitive function. *Epigenomics* 11 401–410. 10.2217/epi-2018-0127 30785307

[B34] Fernandes B. S. Steiner J. Bernstein H. G. Dodd S. Pasco J. A. Dean O. M. (2016). C-reactive protein is increased in schizophrenia but is not altered by antipsychotics: meta-analysis and implications. *Mol. Psychiatry* 21 554–564. 10.1038/mp.2015.87 26169974

[B35] Fillman S. G. Weickert T. W. Lenroot R. K. Catts S. V. Bruggemann J. M. Catts V. S. (2016). Elevated peripheral cytokines characterize a subgroup of people with schizophrenia displaying poor verbal fluency and reduced Broca’s area volume. *Mol. Psychiatry* 21 1090–1098. 10.1038/mp.2015.90 26194183 PMC4960447

[B36] Frank O. Giehl M. Hehlmann R. Zheng C. Leib-Mosch C. Seifarth W. (2005). Human endogenous retrovirus expression profiles in samples from brains of patients with schizophrenia and bipolar disorders. *J. Virol.* 79 10890–10901. 10.1128/jvi.79.17.10890-10901.2005 16103141 PMC1193590

[B37] Frydecka D. Krzystek-Korpacka M. Lubeiro A. Stramecki F. Staǹczykiewicz B. Aleksander Beszłej J. (2018). Profiling inflammatory signatures of schizophrenia: a cross-sectional and meta-analysis study. *Brain Behav. Immun.* 71 28–36. 10.1016/j.bbi.2018.05.002 29730395

[B38] Frydecka D. Misiak B. Pawlak-Adamska E. Karabon L. Tomkiewicz A. Sedlaczek P. (2015). Interleukin-6: the missing element of the neurocognitive deterioration in schizophrenia? The focus on genetic underpinnings, cognitive impairment and clinical manifestation. *Eur. Arch. Psychiatry Clin. Neurosci.* 265 449–459. 10.1007/s00406-014-0533-5 25214388 PMC4540774

[B39] Gentilini D. Mari D. Castaldi D. Remondini D. Ogliari G. Ostan R. (2013). Role of epigenetics in human aging and longevity: genome-wide DNA methylation profile in centenarians and centenarians’ offspring. *Age* 35 1961–1973. 10.1007/s11357-012-9463-1 22923132 PMC3776126

[B40] Goldsmith D. R. Haroon E. Miller A. H. Strauss G. P. Buckley P. F. Miller B. J. (2018). TNF-α and IL-6 are associated with the deficit syndrome and negative symptoms in patients with chronic schizophrenia. *Schizophr. Res.* 199 281–284. 10.1016/j.schres.2018.02.048 29499967 PMC6111000

[B41] Goldsmith D. R. Rapaport M. H. Miller B. J. (2016). A meta-analysis of blood cytokine network alterations in psychiatric patients: comparisons between schizophrenia, bipolar disorder and depression. *Mol. Psychiatry* 199 281–284. 26903267 10.1038/mp.2016.3 PMC6056174

[B42] Grandi N. Tramontano E. (2017). Type W human endogenous retrovirus (HERV-W) integrations and their mobilization by L1 machinery: contribution to the human transcriptome and impact on the host physiopathology. *Viruses* 9:E162. 10.3390/v9070162 28653997 PMC5537654

[B43] Grandi N. Tramontano E. (2018). Human endogenous retroviruses are ancient acquired elements still shaping innate immune responses. *Front. Immunol.* 9:2039. 10.3389/fimmu.2018.02039 30250470 PMC6139349

[B44] Griffiths D. J. (2001). Endogenous retroviruses in the human genome sequence. *Genome Biol.* 2 reviews1017.1–reviews1017.5. 10.1186/gb-2001-2-6-reviews1017 PMC138943 11423012

[B45] Hernández H. G. Mahecha M. F. Mejía A. Arboleda H. Forero D. A. (2014). Global long interspersed nuclear element 1 DNA methylation in a Colombian sample of patients with late-onset Alzheimer’s disease. *Am. J. Alzheimers Dis. Other Demen.* 29 50–53. 10.1177/1533317513505132 24164934 PMC11008131

[B46] Hiltunen M. Helisalmi S. Mannermaa A. Alafuzoff I. Koivisto A. M. Lehtovirta M. (2000). Identification of a novel 4.6-kb genomic deletion in presenilin-1 gene which results in exclusion of exon 9 in a Finnish early onset Alzheimer’s disease family: an Alu core sequence-stimulated recombination? *Eur. J. Hum. Genet.* 8 259–266. 10.1038/sj.ejhg.5200423 10854108

[B47] Houck C. M. Rinehart F. P. Schmid C. W. (1979). A ubiquitous family of repeated DNA sequences in the human genome. *J. Mol. Biol.* 132 289–306. 10.1016/0022-2836(79)90261-4 533893

[B48] Huang W. Li S. Hu Y. Yu H. Luo F. Zhang Q. (2011). Implication of the env gene of the human endogenous retrovirus W family in the expression of BDNF and DRD3 and development of recent-onset schizophrenia. *Schizophr. Bull.* 37 988–1000. 10.1093/schbul/sbp166 20100784 PMC3160218

[B49] Huang W. J. Liu Z. C. Wei W. Wang G. H. Wu J. G. Zhu F. (2006). Human endogenous retroviral pol RNA and protein detected and identified in the blood of individuals with schizophrenia. *Schizophr. Res.* 83 193–199. 10.1016/j.schres.2006.01.007 16531011

[B50] Hurst T. P. Magiorkinis G. (2015). Activation of the innate immune response by endogenous retroviruses. *J. Gen. Virol.* 96 1207–1218. 10.1099/jgv.0.000017 26068187

[B51] Jaworska-Andryszewska P. Rybakowski J. K. (2019). Childhood trauma in mood disorders: neurobiological mechanisms and implications for treatment. *Pharmacol. Rep.* 71 112–120. 10.1016/j.pharep.2018.10.004 30544098

[B52] Jintaridth P. Mutirangura A. (2010). Distinctive patterns of age-dependent hypomethylation in interspersed repetitive sequences. *Physiol. Genomics* 41 194–200. 10.1152/physiolgenomics.00146.2009 20145203

[B53] Jung M. Pfeifer G. P. (2015). Aging and DNA methylation. *BMC Biol.* 13:7. 10.1186/s12915-015-0118-4 25637097 PMC4311512

[B54] Kaer K. Speek M. (2013). Retroelements in human disease. *Gene* 518 231–241. 10.1016/j.gene.2013.01.008 23333607

[B55] Kalayasiri R. Kraijak K. Mutirangura A. Maes M. (2019). Paranoid schizophrenia and methamphetamine-induced paranoia are both characterized by a similar LINE-1 partial methylation profile, which is more pronounced in paranoid schizophrenia. *Schizophr. Res.* 208 221–227. 10.1016/j.schres.2019.02.015 30826260

[B56] Karlsson H. McArthur J. Schroder J. Torrey E. F. Bachmann S. Yolken R. H. (2002). Retroviral RNA identified in the cerebrospinal fluids and brains of individuals with schizophrenia. *Proc. Natl. Acad. Sci. U.S.A.* 98 4634–4639. 10.1073/pnas.061021998 11296294 PMC31886

[B57] Karlsson H. Schröder J. Bachmann S. Bottmer C. Yolken R. H. (2004). HERV-W-related RNA detected in plasma from individuals with recent-onset schizophrenia or schizoaffective disorder. *Mol. Psychiatry* 9 12–13. 10.1038/sj.mp.4001439 14571258

[B58] Karpiǹski P. Frydecka D. Sasiadek M. M. Misiak B. (2016). Reduced number of peripheral natural killer cells in schizophrenia but not in bipolar disorder. *Brain Behav. Immun.* 54 194–200. 10.1016/j.bbi.2016.02.005 26872421

[B59] Karpiǹski P. Samochowiec J. Frydecka D. Sąsiadek M. M. Misiak B. (2018). Further evidence for depletion of peripheral blood natural killer cells in patients with schizophrenia: a computational deconvolution study. *Schizophr. Res.* 54 194–200. 10.1016/j.schres.2018.04.026 29681501

[B60] Kazazian H. H. (2014). Processed pseudogene insertions in somatic cells. *Mob. DNA* 5:20. 10.1186/1759-8753-5-20 25184004 PMC4151081

[B61] Khandaker G. M. Zimbron J. Lewis G. Jones P. B. (2013). Prenatal maternal infection, neurodevelopment and adult schizophrenia: a systematic review of population-based studies. *Psychol. Med.* 43 239–257. 10.1017/S0033291712000736 22717193 PMC3479084

[B62] Kitkumthorn N. Mutirangura A. (2011). Long interspersed nuclear element-1 hypomethylation in cancer: biology and clinical applications. *Clin. Epigenetics* 2 315–330. 10.1007/s13148-011-0032-8 22704344 PMC3365388

[B63] Lander E. S. Linton L. M. Birren B. Nusbaum C. Zody M. C. Baldwin J. (2001). Initial sequencing and analysis of the human genome. *Nature* 409 860–921. 10.1038/35057062 11237011

[B64] Lapp H. E. Hunter R. G. (2016). The dynamic genome: transposons and environmental adaptation in the nervous system. *Epigenomics* 8 237–249. 10.2217/epi.15.107 26791965

[B65] Laska M. J. Troldborg A. Hauge E. M. Bahrami S. Stengaard-Pedersen K. (2017). Human endogenous retroviral genetic element with immunosuppressive activity in both human autoimmune diseases and experimental arthritis. *Arthritis Rheumatol.* 69 398–409. 10.1002/art.39867 27696782

[B66] Li F. Sabunciyan S. Yolken R. H. Lee D. Kim S. Karlsson H. (2019). Transcription of human endogenous retroviruses in human brain by RNA-seq analysis. *PLoS One* 14:e0207353. 10.1371/journal.pone.0207353 30605476 PMC6317784

[B67] Li S. Yang Q. Hou Y. Jiang T. Zong L. Wang Z. (2018). Hypomethylation of LINE-1 elements in schizophrenia and bipolar disorder. *J. Psychiatr. Res.* 107 68–72. 10.1016/j.jpsychires.2018.10.009 30326341

[B68] Li T. H. Schmid C. W. (2001). Differential stress induction of individual Alu loci: implications for transcription and retrotransposition. *Gene* 276 135–141. 10.1016/s0378-1119(01)00637-0 11591480

[B69] Mak M. Samochowiec J. Frydecka D. Pełka-Wysiecka J. Szmida E. Karpiǹski P. (2019). First-episode schizophrenia is associated with a reduction of HERV-K methylation in peripheral blood. *Psychiatry Res.* 271 459–463. 10.1016/j.psychres.2018.12.012 30537669

[B70] Mameli G. Astone V. Arru G. Marconi S. Lovato L. Serra C. (2007). Brains and peripheral blood mononuclear cells of multiple sclerosis (MS) patients hyperexpress MS-associated retrovirus/HERV-W endogenous retrovirus, but not human herpesvirus 6. *J. Gen. Virol.* 88 264–274. 10.1099/vir.0.81890-0 17170460

[B71] Marangoni C. Hernandez M. Faedda G. L. (2016). The role of environmental exposures as risk factors for bipolar disorder: a systematic review of longitudinal studies. *J. Affect. Disord.* 193 165–174. 10.1016/j.jad.2015.12.055 26773919

[B72] McGrath J. J. Welham J. L. (1999). Season of birth and schizophrenia: a systematic review and meta-analysis of data from the Southern Hemisphere. *Schizophr. Res.* 35 237–242. 10.1016/s0920-9964(98)00139-x 10093868

[B73] McGuffin P. Rijsdijk F. Andrew M. Sham P. Katz R. Cardno A. (2003). The heritability of bipolar affective disorder and the genetic relationship to unipolar depression. *Arch. Gen. Psychiatry* 60 497–502. 12742871 10.1001/archpsyc.60.5.497

[B74] Melbourne J. K. Chase K. A. Feiner B. Rosen C. Sharma R. P. (2018). Long non-coding and endogenous retroviral RNA levels are associated with proinflammatory cytokine mRNA expression in peripheral blood cells: implications for schizophrenia. *Psychiatry Res.* 262 465–468. 10.1016/j.psychres.2017.09.025 28942956 PMC5851803

[B75] Meredith R. M. (2015). Sensitive and critical periods during neurotypical and aberrant neurodevelopment: a framework for neurodevelopmental disorders. *Neurosci. Biobehav. Rev.* 50 180–188. 10.1016/j.neubiorev.2014.12.001 25496903

[B76] Mi S. Lee X. Li X. Veldman G. M. Finnerty H. Racie L. (2000). Syncytin is a captive retroviral envelope protein involved in human placental morphogenesis. *Nature* 403 785–789. 10.1038/35001608 10693809

[B77] Mighdoll M. I. Tao R. Kleinman J. E. Hyde T. M. (2015). Myelin, myelin-related disorders, and psychosis. *Schizophr. Res.* 161 85–93. 10.1016/j.schres.2014.09.040 25449713

[B78] Mighell A. J. Markham A. F. Robinson P. A. (1997). Alu sequences. *FEBS Lett.* 417 1–5. 10.1016/s0014-5793(97)01259-3 9395063

[B79] Miglino N. Tamm M. Borger P. (2014). Transposable element LINE1 is activated after exposure to cigarette smoke in primary human lung fibroblasts. *Eur. Respir. J.* 44:S2023.

[B80] Miller B. J. Buckley P. Seabolt W. Mellor A. Kirkpatrick B. (2011). Meta-analysis of cytokine alterations in schizophrenia: clinical status and antipsychotic effects. *Biol. Psychiatry* 70 663–671. 10.1016/j.biopsych.2011.04.013 21641581 PMC4071300

[B81] Miller B. J. Gassama B. Sebastian D. Buckley P. Mellor A. (2013). Meta-analysis of lymphocytes in schizophrenia: clinical status and antipsychotic effects. *Biol. Psychiatry* 73 993–999. 10.1016/j.biopsych.2012.09.007 23062357 PMC3816144

[B82] Milne T. A. McEwen B. S. Hunter R. G. Pfaff D. W. McCarthy K. J. (2009). Regulation of hippocampal H3 histone methylation by acute and chronic stress. *Proc. Natl. Acad. Sci. U.S.A.* 106 20912–20917. 10.1073/pnas.0911143106 19934035 PMC2791599

[B83] Miousse I. R. Chalbot M. C. G. Lumen A. Ferguson A. Kavouras I. G. Koturbash I. (2015). Response of transposable elements to environmental stressors. *Mutat. Res. Rev. Mutat. Res.* 765 19–39. 10.1016/j.mrrev.2015.05.003 26281766 PMC4544780

[B84] Misiak B. Frydecka D. Piotrowski P. Kiejna A. (2013). The multidimensional nature of metabolic syndrome in schizophrenia: lessons from studies of one-carbon metabolism and DNA methylation. *Epigenomics* 5 317–329. 10.2217/epi.13.22 23750646

[B85] Misiak B. Frydecka D. Rybakowski J. K. (2016). Editorial: endophenotypes for schizophrenia and mood disorders: implications from genetic, biochemical, cognitive, behavioral, and neuroimaging studies. *Front. Psychiatry* 7:83. 10.3389/fpsyt.2016.00083 27242553 PMC4862973

[B86] Misiak B. Frydecka D. Staǹczykiewicz B. Samochowiec J. (2019). Editorial: peripheral markers of immune response in major psychiatric disorders: where are we now and where do we want to be? *Front. Psychiatry* 10:5. 10.3389/fpsyt.2019.00005 30723427 PMC6349819

[B87] Misiak B. Krefft M. Bielawski T. Moustafa A. A. Sąsiadek M. M. Frydecka D. (2017a). Toward a unified theory of childhood trauma and psychosis: a comprehensive review of epidemiological, clinical, neuropsychological and biological findings. *Neurosci. Biobehav. Rev.* 75 393–406. 10.1016/j.neubiorev.2017.02.015 28216171

[B88] Misiak B. Staǹczykiewicz B. Kotowicz K. Rybakowski J. K. Samochowiec J. Frydecka D. (2017b). Cytokines and C-reactive protein alterations with respect to cognitive impairment in schizophrenia and bipolar disorder: a systematic review. *Schizophr. Res.* 192 16–29. 10.1016/j.schres.2017.04.015 28416092

[B89] Misiak B. Szmida E. Karpiǹski P. Loska O. Sąsiadek M. M. Frydecka D. (2015). Lower LINE-1 methylation in first-episode schizophrenia patients with the history of childhood trauma. *Epigenomics* 7 1275–1285. 10.2217/epi.15.68 26212695

[B90] Mondelli V. Ciufolini S. Murri M. B. Bonaccorso S. Di Forti M. Giordano A. (2015). Cortisol and inflammatory biomarkers predict poor treatment response in first episode psychosis. *Schizophr. Bull.* 41 1162–1170. 10.1093/schbul/sbv028 25829375 PMC4535637

[B91] Morozov V. A. Dao Thi V. L. Denner J. (2013). The transmembrane protein of the human endogenous retrovirus - K (HERV-K) modulates cytokine release and gene expression. *PLoS One* 8:e70399. 10.1371/journal.pone.0070399 23950929 PMC3737193

[B92] Mortensen P. B. Pedersen C. B. Mcgrath J. J. Hougaard D. M. Nørgaard-Petersen B. Mors O. (2011). Neonatal antibodies to infectious agents and risk of bipolar disorder: a population-based case-control study. *Bipolar Disord.* 13 624–629. 10.1111/j.1399-5618.2011.00962.x 22085475

[B93] Munoz-Lopez M. Garcia-Perez J. (2010). DNA transposons: nature and applications in genomics. *Curr. Genomics* 11 115–128. 10.2174/138920210790886871 20885819 PMC2874221

[B94] Muotri A. R. Chu V. T. Marchetto M. C. N. Deng W. Moran J. V. Gage F. H. (2005). Somatic mosaicism in neuronal precursor cells mediated by L1 retrotransposition. *Nature* 435 903–910. 10.1038/nature03663 15959507

[B95] Muotri A. R. Marchetto M. C. N. Coufal N. G. Oefner R. Yeo G. Nakashima K. (2010). L1 retrotransposition in neurons is modulated by MeCP2. *Nature* 468 443–446. 10.1038/nature09544 21085180 PMC3059197

[B96] Murphy S. K. Fineberg A. M. Maxwell S. D. Alloy L. B. Zimmermann L. Krigbaum N. Y. (2017). Maternal infection and stress during pregnancy and depressive symptoms in adolescent offspring. *Psychiatry Res.* 257 102–110. 10.1016/j.psychres.2017.07.025 28750213 PMC5823248

[B97] Mutirangura A. (ed). (2019). “A hypothesis to explain how the DNA of elderly people is prone to damage: genome-wide hypomethylation drives genomic instability in the elderly by reducing youth-associated gnome-stabilizing DNA gaps,” in *Epigenetics [Working Title]*, (London: Intechopen). 10.5772/intechopen.83372

[B98] Nagy C. Turecki G. (2012). Sensitive periods in epigenetics: bringing us closer to complex behavioral phenotypes. *Epigenomics* 4 445–457. 10.2217/epi.12.37 22920183 PMC5293543

[B99] Najjar S. Pearlman D. M. (2015). Neuroinflammation and white matter pathology in schizophrenia: systematic review. *Schizophr. Res.* 161 102–112. 10.1016/j.schres.2014.04.041 24948485

[B100] Nestler E. J. Peña C. J. Kundakovic M. Mitchell A. Akbarian S. (2016). Epigenetic basis of mental illness. *Neuroscientist* 22 447–463. 10.1177/1073858415608147 26450593 PMC4826318

[B101] Oja M. Peltonen J. Blomberg J. Kaski S. (2008). Methods for estimating human endogenous retrovirus activities from EST databases. *BMC Bioinformatics* 8(Suppl. 2):S11. 10.1186/1471-2105-8-S2-S11 17493249 PMC1892069

[B102] Ostertag E. M. Goodier J. L. Zhang Y. Kazazian H. H. (2003). SVA elements are nonautonomous retrotransposons that cause disease in humans. *Am. J. Hum. Genet.* 73 1444–1451. 10.1086/380207 14628287 PMC1180407

[B103] Parboosing R. Bao Y. Shen L. Schaefer C. A. Brown A. S. (2013). Gestational influenza and bipolar disorder in adult offspring. *JAMA Psychiatry* 70 677–685. 10.1001/jamapsychiatry.2013.896 23699867

[B104] Patchsung M. Settayanon S. Pongpanich M. Mutirangura D. Jintarith P. Mutirangura A. (2018). Alu siRNA to increase Alu element methylation and prevent DNA damage. *Epigenomics* 10 175–185. 10.2217/epi-2017-0096 29336607

[B105] Perron H. Hamdani N. Faucard R. Lajnef M. Jamain S. Daban-Huard C. (2012). Molecular characteristics of Human Endogenous Retrovirus type-W in schizophrenia and bipolar disorder. *Transl. Psychiatry* 2:e201. 10.1038/tp.2012.125 23212585 PMC3565190

[B106] Perron H. Mekaoui L. Bernard C. Veas F. Stefas I. Leboyer M. (2008). Endogenous retrovirus type W GAG and envelope protein antigenemia in serum of schizophrenic patients. *Biol. Psychiatry* 64 1019–1023. 10.1016/j.biopsych.2008.06.028 18760403

[B107] Poduri A. Evrony G. D. Cai X. Walsh C. A. (2013). Somatic mutation, genomic variation, and neurological disease. *Science* 341:1237758. 10.1126/science.1237758 23828942 PMC3909954

[B108] Pray L. A. (2008a). Functions and utility of alu jumping genes. *Nature* 1:93. 25974184

[B109] Pray L. A. (2008b). Transposons: the jumping genes. *Nat. Educ.* 1:204.

[B110] Protasova M. S. Gusev F. E. Grigorenko A. P. Kuznetsova I. L. Rogaev E. I. Andreeva T. V. (2017). Quantitative analysis of L1-retrotransposons in Alzheimer’s disease and aging. *Biochemistry* 82 962–971. 10.1134/S0006297917080120 28941465

[B111] Ripke S. Neale B. M. Corvin A. Walters J. T. R. Farh K. H. Holmans P. A. (2014). Biological insights from 108 schizophrenia-associated genetic loci. *Nature* 511 421–427. 10.1038/nature13595 25056061 PMC4112379

[B112] Rusiecki J. A. Chen L. Srikantan V. Zhang L. Yan L. Polin M. L. (2012). DNA methylation in repetitive elements and post-traumatic stress disorder: a case-control study of US military service members. *Epigenomics* 4 29–40. 10.2217/epi.11.116 22332656 PMC3809831

[B113] Saeliw T. Tangsuwansri C. Thongkorn S. Chonchaiya W. Suphapeetiporn K. Mutirangura A. (2018). Integrated genome-wide Alu methylation and transcriptome profiling analyses reveal novel epigenetic regulatory networks associated with autism spectrum disorder. *Mol. Autism* 9:27. 10.1186/s13229-018-0213-9 29686828 PMC5902935

[B114] Sassaman D. M. Dombroski B. A. Moran J. V. Kimberland M. L. Naas T. P. DeBerardinis R. J. (1997). Many human L1 elements are capable of retrotransposition. *Nat. Genet.* 16 37–43. 10.1038/ng0597-37 9140393

[B115] Schumann G. G. Gogvadze E. V. Osanai-Futahashi M. Kuroki A. Münk C. Fujiwara H. (2010). Unique functions of repetitive transcriptomes. *Int. Rev. Cell Mol. Biol.* 285 115–188. 10.1016/B978-0-12-381047-2.00003-7 21035099

[B116] Shpyleva S. Melnyk S. Pavliv O. Pogribny I. Jill James S. (2018). Overexpression of LINE-1 retrotransposons in autism brain. *Mol. Neurobiol.* 55 1740–1749. 10.1007/s12035-017-0421-x 28220356

[B117] Su J. Shao X. Liu H. Liu S. Wu Q. Zhang Y. (2012). Genome-wide dynamic changes of DNA methylation of repetitive elements in human embryonic stem cells and fetal fibroblasts. *Genomics* 99 10–17. 10.1016/j.ygeno.2011.10.004 22044633

[B118] Suarez N. A. Macia A. Muotri A. R. (2018). LINE-1 retrotransposons in healthy and diseased human brain. *Dev. Neurobiol.* 78 434–455. 10.1002/dneu.22567 29239145 PMC5897138

[B119] Talens R. P. Christensen K. Putter H. Willemsen G. Christiansen L. Kremer D. (2012). Epigenetic variation during the adult lifespan: cross-sectional and longitudinal data on monozygotic twin pairs. *Aging Cell* 11 694–703. 10.1111/j.1474-9726.2012.00835.x 22621408 PMC3399918

[B120] Tangsuwansri C. Saeliw T. Thongkorn S. Chonchaiya W. Suphapeetiporn K. Mutirangura A. (2018). Investigation of epigenetic regulatory networks associated with autism spectrum disorder (ASD) by integrated global LINE-1 methylation and gene expression profiling analyses. *PLoS One* 13:e0201071. 10.1371/journal.pone.0201071 30036398 PMC6056057

[B121] Tartaglione A. M. Cipriani C. Chiarotti F. Perrone B. Balestrieri E. Matteucci C. (2018). Early behavioral alterations and increased expression of endogenous retroviruses are inherited across generations in mice prenatally exposed to valproic acid. *Mol. Neurobiol.* 56 3736–3750. 10.1007/s12035-018-1328-x 30194517

[B122] Van Den Oord E. J. C. G. Clark S. L. Xie L. Y. Shabalin A. A. Dozmorov M. G. Kumar G. (2016). A whole methylome CpG-SNP association study of psychosis in blood and brain tissue. *Schizophr. Bull.* 42 1018–1026. 10.1093/schbul/sbv182 26656881 PMC4903046

[B123] Vargiu L. Rodriguez-Tomé P. Sperber G. O. Cadeddu M. Grandi N. Blikstad V. (2016). Classification and characterization of human endogenous retroviruses mosaic forms are common. *Retrovirology* 13:7. 10.1186/s12977-015-0232-y 26800882 PMC4724089

[B124] Vidaud D. Vidaud M. Bahnak B. R. Siguret V. Gispert Sanchez S. Laurian Y. (1993). Haemophilia B due to a de novo insertion of a human-specific Alu subfamily member within the coding region of the factor IX gene. *Eur. J. Hum. Genet.* 1 30–36. 8069649 10.1159/000472385

[B125] Vigo D. Thornicroft G. Atun R. (2016). Estimating the true global burden of mental illness. *Lancet Psychiatry* 3 171–178. 10.1016/S2215-0366(15)00505-2 26851330

[B126] Wang H. Xing J. Grover D. Hedges Kyudong Han D. J. Walker J. A. (2005). SVA elements: a hominid-specific retroposon family. *J. Mol. Biol.* 354 994–1007. 10.1016/j.jmb.2005.09.085 16288912

[B127] Weis S. Llenos I. C. Sabunciyan S. Dulay J. R. Isler L. Yolken R. (2007). Reduced expression of human endogenous retrovirus (HERV)-W GAG protein in the cingulate gyrus and hippocampus in schizophrenia, bipolar disorder, and depression. *J. Neural Transm.* 114 645–655. 10.1007/s00702-006-0599-y 17219017

[B128] Yao Y. Schröder J. Nellåker C. Bottmer C. Bachmann S. Yolken R. H. (2008). Elevated levels of human endogenous retrovirus-W transcripts in blood cells from patients with first episode schizophrenia. *Genes Brain Behav.* 7 103–112. 17559415 10.1111/j.1601-183X.2007.00334.x

[B129] Zhang Z. Harrison P. M. Liu Y. Gerstein M. (2003). Millions of years of evolution preserved: a comprehensive catalog of the processed pseudogenes in the human genome. *Genome Res.* 13 2541–2558. 10.1101/gr.1429003 14656962 PMC403796

[B130] Zhao K. Du J. Peng Y. Li P. Wang S. Wang Y. (2018). LINE1 contributes to autoimmunity through both RIG-I- and MDA5-mediated RNA sensing pathways. *J. Autoimmun.* 90 105–115. 10.1016/j.jaut.2018.02.007 29525183

